# Adsorption of Welan Gum on Montmorillonite and Its Influencing Factors

**DOI:** 10.3390/polym14132599

**Published:** 2022-06-27

**Authors:** Kaili Liao, Junnan An, Lipei Fu, Houye Zhang, Meng Wei, Jinmei Bai, Yanfeng He

**Affiliations:** 1 School of Petroleum Engineering, Changzhou University, Changzhou 213164, China; liaokaili@cczu.edu.cn (K.L.); 20085700158@smail.cczu.edu.cn (J.A.); s21040857008@smail.cczu.edu.cn (H.Z.); s21040857036@smail.cczu.edu.cn (M.W.); baijinmei@cczu.edu.cn (J.B.); 2 School of Overseas Education, Changzhou University, Changzhou 213164, China

**Keywords:** welan gum, polymer flooding, enhancing oil recovery, montmorillonite, adsorption

## Abstract

Welan gum is one of the most promising polymers used in polymer flooding for enhancing oil recovery, due to its excellent temperature resistance and salt-tolerance performance. However, welan gum, as a polymer with higher molecular weight, can be adsorbed and detained in the pore throat of the reservoir, which is characterized by a smaller size. Montmorillonite, a kind of clay mineral with high content in reservoir rocks, has strong adsorption capacity. Therefore, the adsorption behavior of welan gum on montmorillonite, as well as its influencing factors, are studied in this paper. The results show that the adsorption capacity is 2.07 mg/g. The adsorption capacity decreased with the increase in temperature. Both acidic and alkaline conditions reduced the adsorption capacity. The existence of inorganic salt affected the adsorption capacity. In addition, the higher the cation value, the lower the adsorption capacity. The characterization tests showed that the adsorption of welan gum on montmorillonite was characterized by physical adsorption and surface adsorption, indicating that there were no changes in the internal structure of montmorillonite. This study provides feasible methods to reduce the amount of welan gum adsorbed on montmorillonite, which is of great significance for reducing the permeability damage caused by welan gum adsorption and promoting the application of welan gum in polymer flooding for enhancing oil recovery.

## 1. Introduction

With the development of the global economy, the demand for oil and gas is increasing year by year. In order to increase the production of crude oil, many countries have shifted the focus of oil exploration and development to oil and gas resources in reservoirs with high temperatures and high salt content [1]. As the main chemical flooding technology, polymer flooding has a good oil displacement effect in conventional reservoirs and can greatly improve oil recovery [2,3]. However, in a high-temperature, high-salt environment, commonly used polymers, such as partially hydrolyzed polyacrylamide (HPAM), are easily degraded, resulting in viscosity loss and oil recovery reduction [4,5].

In addition, because polyvinyl alcohol (PVA) has good thermal stability, electrolytic stability, and an interfacial tension reduction ability, some scholars believe it has better application prospects in high-temperature, high-salt reservoirs [6,7]. Therefore, the preparation of modified polyvinyl alcohol and its application evaluation in oil displacement were also carried out [8,9]. However, in general, the research on polymer flooding in high-temperature, high-salt reservoirs is still dominated by HPAM, that is, by modifying the polymer to improve its temperature resistance and salt tolerance. Gou et al. improved the rheological properties of HPAM by introducing a sulfonate, a hydrophobic long chain, and a ring structure into the acrylamide polymer chain [10]. Zhang et al. found that the copolymer had a better oil recovery rate (15.43%) than the common HPAM (7.41%) [11].

**Figure 1:**
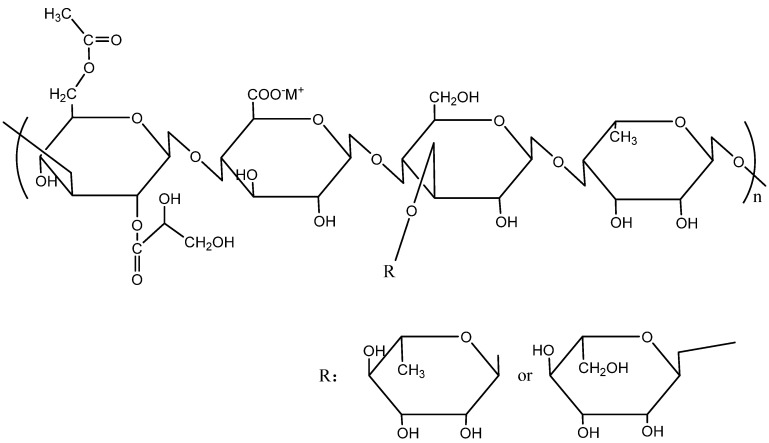
Molecular structure of welan gum.

For the above problems of polymers, some scholars have found that microbial polysaccharide had good viscosity-enhancement ability and excellent temperature resistance/salt-tolerance performance compared with HPAM [12]. Typical studies of microbial polysaccharide used in polymer flooding in recent years are shown in Table 1. Li et al. pointed out that xanthan gum exhibited better viscosity-retention ability than HPAM when the temperature was 80 °C however, its viscosity loss became greater if the temperature was higher than 100 °C [13]. Xanthan gum and HPAM have synergistic effect in increasing viscosity, when the xanthan gum was more than 80% [14]. Viscosity is affected little by acid and the base environment, and it can be kept stable for 3 days without bactericide [15]. Xu et al. found that xanthan gum could enhance the recovery of heavy oil, because of its good salt-tolerance performance 13. Considering that xanthan gum is not resistant to high temperatures, some scholars turned to another kind of microbial polysaccharide—welan gum. Its molecular structure is shown in Figure 1 [16]. Xu et al. found that welan gum had better viscosity-retention rate (72%) than xanthan gum (50%) at 75 °C, compared with that at the temperature of 25 °C [17]. The enhanced oil recovery rate of welan gum (24.6%) was higher than that of xanthan gum (21.8%) [18]. The good performance of welan gum is closely related to its structure [19]. Welan gum solution is a kind of typical pseudoplastic fluid with good viscoelasticity. It could maintain its viscosity in the presence of sodium ion and potassium ion. Sand-pack flooding tests showed higher displacement efficiency than HPAM [20]. Therefore, welan gum solution has a unique property of maintaining stable viscosity at high temperatures (150 °C) in a wide range of pH values (pH = 2–12), making it a good candidate for use in reservoirs with high temperatures and high salt content [21,22].

For polymer flooding, when the molecular weight is large, the polymer may stay at the reservoir pore throat, blocking the channel for oil and gas migration and harming the reservoir permeability [23,24]. Furthermore, the retention amount may increase in reservoirs with high salinity or in the presence of multivalent cations [24,25]. In addition, Xu et al. found that the retention amount of HPAM increased with the increase in the content of clay minerals [26]. 

There are many reasons for polymer retention in reservoirs, including polymer adsorption, mechanical retention, and hydrodynamic retention [27]. Among them, the damage caused by the adsorption of clay minerals is difficult to remove [28]. Quezada et al. found that the hydrogen bond between HPAM and quartz sand promoted the increase in the adsorption capacity, while the adsorption capacity decreased significantly with the increase in inorganic salt content [29]. Chiappa et al. found that the existence of cations had a great influence on anionic HPAM [30]. Yin et al. studied the adsorption law of hydroxypropyl guanidine gum (which is kind of biopolymer) on clay minerals (kaolin and montmorillonite), and pointed out that the adsorption between hydroxypropyl guanidine gum and clay minerals was physical adsorption, and the adsorption capacity was greatly reduced in acidic conditions or in high-salt conditions [31,32].

Although the molecular weight of welan gum is smaller than that of HPAM and xanthan gum, it can provide a stronger viscosity-increasing performance and better temperature resistance, meaning it has good application prospects in polymer flooding technologies. However, as macromolecular material, welan gum also has retention problems in the reservoir pore and throat. This affects the porosity and permeability of porous media, hinders the migration of oil and gas, and reduces the hydrocarbon production. So, it is necessary to clarify the adsorption law of welan gum on reservoir rocks. As for the adsorption and retention of welan gum on reservoir rocks, Fu et al. studied its adsorption on quartz sand [33]. As another major component of reservoir rocks, montmorillonite is a common clay mineral in reservoir, but the adsorption of welan gum on montmorillonite has not been reported yet. Therefore, this paper studied the adsorption law of welan gum on montmorillonite, and systematically studies its influencing factors in order to explore methods of reducing the adsorption amount. The results have good practical significance for promoting the application of microbial polysaccharide polymers in the petroleum industry.

## 2. Experimental Section

### 2.1. Materials

Welan gum, with a purity of 98%, was purchased from Hebei Xinhe Biochemical Co., Ltd., China. Montmorillonite, with a purity of 98%, was purchased from Aladdin Reagent Co., Ltd., Shanghai, China. NaCl, KCl, MgCl_2_, CaCl_2_, NaOH, and anthrone were analytical grade, and were purchased from Sinopharm, China. Sulfuric acid, with the purity of 98%, was purchased from China National Pharmaceutical Group Corporation, Beijing, China. The ethyl acetate, with the purity of 99%, was purchased from Macklin Biochemical Co., Ltd., Shanghai, China.

### 2.2. Methods

#### 2.2.1. Determination of the Standard Curve of Absorbance–Welan Gum Concentration

**Figure 2:**
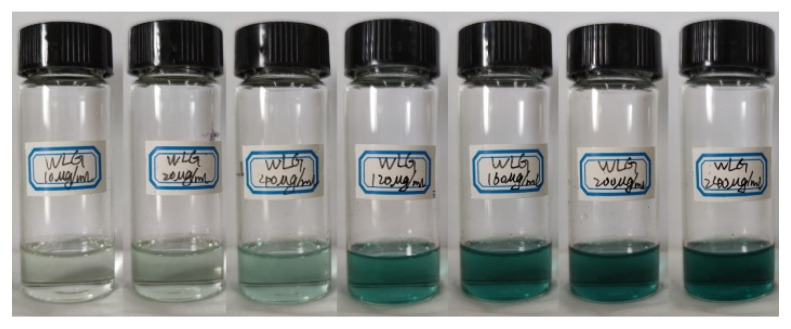
Welan gum solutions with different concentrations (from left to right: 10 μg/mL, 20 μg/mL, 40 μg/mL, 80 μg/mL, 120 μg/mL, 160 μg/mL, 200 μg/mL, and 240 μg/mL) after chromogenic reaction.

**Figure 3:**
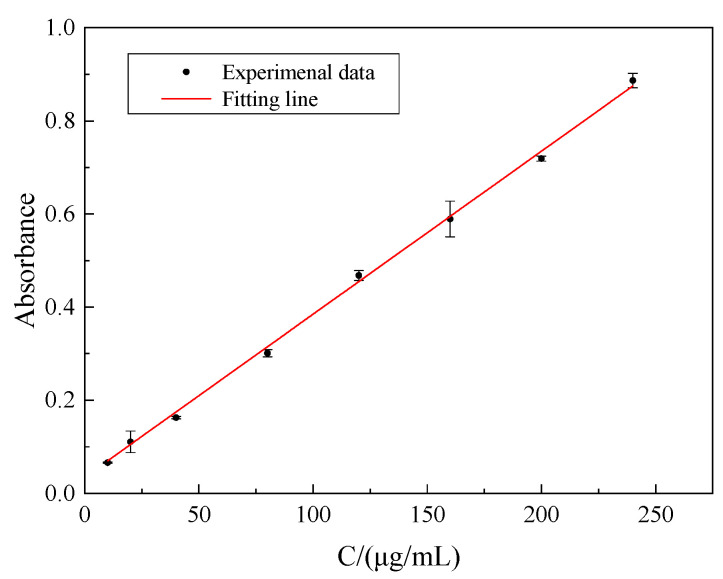
Standard curve of the absorbance–concentration of welan gum solution.

The establishment of the standard curve that reflects the absorbance and the concentration of welan gum, has four steps: (1) a series of welan gum solutions with different concentrations (10 μg/mL, 20 μg/mL, 40 μg/mL, 80 μg/mL, 120 μg/mL, 160 μg/mL, 200 μg/mL, and 240 μg/mL), and the ethyl acetate solution of anthrone with the concentration of 2 mg/mL, were prepared; (2) 1 mL of the ethyl acetate solution of anthrone and 3 mL of the sulfuric acid were added into 2 mL of the welan gum solution. The mixture was placed in a water bath of 85 °C for 10 min. The mixture was taken out and placed in a 25 °C environment for 14 min. Then, the photo shown in Figure 2 was taken; (3) the absorbance of the above solutions was measured by a UV–vis spectrophotometer (Shanghai Instrument Co., Ltd., 752 N, Shanghai, China); (4) the relationship curve between the concentration and absorbance was drawn, which is called the standard curve, as shown in Figure 3.

The principle of determining the standard curve is as follows. The reaction between welan gum with sulfuric acid produce hydroxymethyl furfural. This product can further react with anthrone to form furfural derivatives, which are a blue-green color. Therefore, the color of the solution changes after the reaction. Within a certain concentration range, the color of the solution from light to dark is proportional to the content of welan gum. Since the absorption peak of the above furfural derivatives is 630 nm in the visible region, the depth of the color can be determined by the absorbance value measured by the spectrophotometer. Therefore, the content of welan gum in the solution can be determined by the absorbance value. Furthermore, the standard curve representing the relationship between the absorbance and the concentration of the welan gum solution can be obtained by performing the above analysis on the welan gum solutions of different concentrations.

#### 2.2.2. Determination of Adsorption Capacity

Montmorillonite (*m* = 1 g) was added into the welan gum solution (*V* = 50 mL). In order to ensure the welan gum was fully adsorbed on the montmorillonite, the mixture was placed in the thermostatic water bath oscillator (Changzhou Tianrui Instrument Co., Ltd., SHA-A, Changzhou, China) and shook for different times according to the experimental requirements. Then, the samples were centrifuged. A measure of 2 mL of the supernatant was taken out and put into a sample bottle for chromogenic reaction. After that, the absorbance was measured by using the UV-vis spectrophotometer, and the concentration (*C*_2_) of welan gum solution after the adsorption test can be obtained from the standard curve. Based on this, the adsorption capacity can be calculated according to the following formula.

(1) $$\Gamma =\frac{(C_{1}-C_{2})V}{1000\times m}$$

where *Γ* is the adsorption capacity, mg/g; *C*_1_ and *C*_2_ are the concentrations of welan gum solution before and after adsorption, respectively, μg/mL; *V* is the volume of welan gum solution, mL; *m* is the mass of montmorillonite, g.

#### 2.2.3. Evaluation of the Effect of Temperature, pH Value, and Inorganic Salt on Adsorption Performance

(1) Determination of the effect of temperature:

A welan gum solution with a concentration of 240 μg/mL was prepared. The solution was divided into 21 parts, and the volume of each sample was 50 mL. 

A measure of 1 g of the montmorillonite was added into each solution, respectively. The solid–liquid mixtures were put into the thermostatic water bath oscillator with oscillation of 240 r/min. The treating time was set as 10 min for the welan gum to be fully adsorbed on montmorillonite. The temperature was set as 25 °C, 30 °C, 40 °C, 50 °C, 60 °C, 70 °C, and 80 °C, respectively. At each temperature, three tests were conducted, and the average was taken as the final result. 

In this group of experiments, no inorganic salt was added into the welan gum solution, and the pH value of the welan gum solution was adjusted to 7 by using diluted hydrochloric acid solution or sodium hydroxide solution.

(2) Determination of the effect of pH value:

A welan gum solution with a concentration of 240 μg/mL was prepared. The solution was divided into 30 parts, and the volume of each solution sample was 50 mL. The diluted hydrochloric acid solution or sodium hydroxide solution were used to adjust the pH value of the above welan gum solution to 3–12 (3, 4, 5, 6, 7, 8, 9, 10, 11, and 12), respectively, and there were three solution samples for each pH value. 

A measure of 1 g of the montmorillonite was added into each solution, respectively. The solid–liquid mixtures were put into the thermostatic water bath oscillator with oscillation of 240 r/min. The treating time was 10 min. 

In this group of experiments, the temperature was 25 °C, and no inorganic salt was added into the welan gum solution. Three tests were performed to obtain the pH value and the average was taken as the final result.

(3) Determination of the effect of inorganic salt:

A welan gum solution with a concentration of 240 μg/mL was prepared. The solution was divided into 30 parts, and the volume of each solution sample was 50 mL. A certain amount of NaCl was added into the solution samples to obtain solutions with the NaCl concentration of 0.05 mol/L, 0.1 mol/L, 0.15 mol/L, 0.2 mol/L, 0.25 mol/L, and 0.3 mol/L. For each concentration of NaCl, three solutions were prepared for control testing. Now, a total of 18 samples of welan gum solution containing NaCl were prepared. Using the same method, 18 replicates of welan gum solutions containing different concentrations of KCl (or MgCl_2_, or CaCl_2_) were prepared.

In this group of experiments, the temperature was 25 °C, and the pH value of the welan gum solution was adjusted to 7 by using diluted hydrochloric acid solution or sodium hydroxide solution. The tests for each inorganic salt at different concentrations were repeated three times, and the average was taken as the final result.

## 3. Results and Discussion

### 3.1. Adsorption Behavior

(1) Standard curve of absorbance–welan concentration:

The chromogenic reactions of welan gum solutions with different concentrations were carried out. The absorbance at the wavelength of 630 nm was measured through the UV–vis spectrophotometer, and the relationship between the absorbance and the concentration of welan gum was obtained. These points were linearly fitted, and the results are shown in Figure 3.

It can be found in Figure 3 that the mathematical expression of the relationship between the absorbance and the concentration of welan gum solution can be obtained as follows:

*Y* = 0.0035*X* − 0.03 (2)

where *Y* is the absorbance, dimensionless; *X* is the concentration of welan gum solution, μg/mL. 

The correlation coefficient is 0.9986, indicating that the absorbance has a good linear relationship with the concentration of welan gum solution. Therefore, this curve can be considered as the standard curve of the relationship between the absorbance and the concentration of welan gum solution.

**Figure 4:**
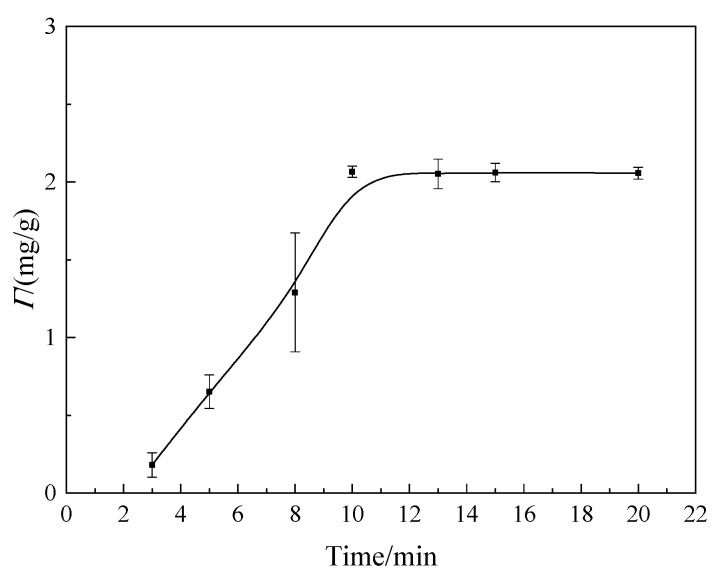
Relationship between the adsorption capacity and the adsorption time.

**Figure 5:**
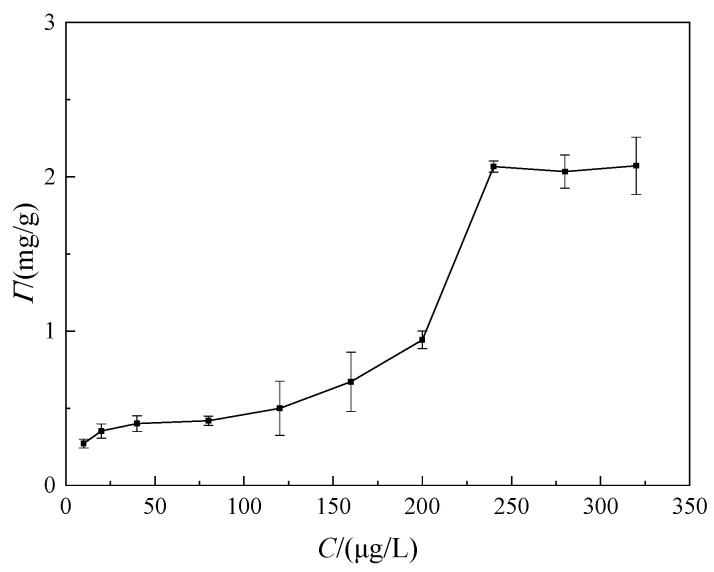
Relationship between the adsorption capacity and the concentration of welan gum solution.

(2) Adsorption equilibrium state:

In order to investigate the adsorption equilibrium state, the effect of the adsorption time and the concentration of welan gum solution on the adsorption capacity were studied. The results are shown in Figure 4 and Figure 5.

In Figure 4, the concentration of the welan gum solutions used in this group of tests was 240 μg/mL. It can be seen from Figure 3 that the adsorption capacity increased at the beginning and then tended to be stable with the increase in time. When the time exceeded 10 min, the adsorption capacity remained almost constant. Therefore, the adsorption equilibrium time for the adsorption of welan gum on montmorillonite was 10 min. 

**Figure 6:**
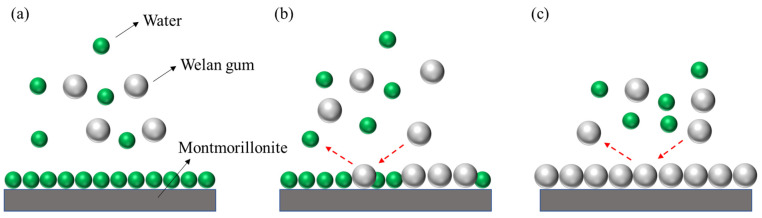
Schematic diagram of the adsorption process and mechanism of welan gum on montmorillonite. (**a**) Before adsorption; (**b**) during adsorption; (**c**) after adsorption.

In Figure 5, the adsorption time for each test was 10 min. As can be seen from Figure 4, with the increase in the concentration of welan gum solution, the adsorption capacity of welan gum on montmorillonite increased. When the concentration increased to 240 μg/mL, the adsorption capacity tended to be stable. For the montmorillonite molecules in solution, its surface was covered by water molecules, as shown in Figure 6a. The adsorption of welan gum on the surface of montmorillonite included two stages. (1) Welan gum molecules gradually replaced water molecules on the surface of montmorillonite, as shown in Figure 6b. Welan gum was easily adsorbed on the surface of montmorillonite due to the hydrogen bond between the active sites on the montmorillonite surface and the welan gum molecules. Its typical characteristic is that the adsorption capacity increased with the increase in welan gum solution concentration. This is because when the concentration was low, there was not enough welan gum in the solution that could be adsorbed, while with the increase in concentration, more and more welan gum could be adsorbed on the montmorillonite. (2) Free welan gum molecules in the solution replaced the adsorbed welan gum molecules on the surface of montmorillonite, which formed a dynamic adsorption–desorption process, as shown in Figure 6c. Its typical characteristic is that the adsorption capacity did not continue to increase once the solution concentration reached a certain value. The reason is that adsorption is a dynamic process—that is, while welan gum was adsorbed onto the surface of montmorillonite, it was also desorbed from the surface of the montmorillonite. If the adsorption amount was equal to the desorption amount in a period of time, then a dynamic equilibrium state was reached. At this time, the macroscopic result is that the adsorption capacity did not continue to increase. In addition, it can be seen from the adsorption isotherm that it was an L-shaped curve, which conforms to the Langmuir adsorption model. It shows that the adsorption of welan gum on montmorillonite is a monolayer adsorption, as shown in Figure 6. This also explains the reason that the adsorption amount of welan gum on montmorillonite gradually tends to be balanced. The saturated adsorption capacity can be calculated as 2.07 mg/g, according to the calculating formula.

### 3.2. Adsorption Mechanism

**Figure 7:**
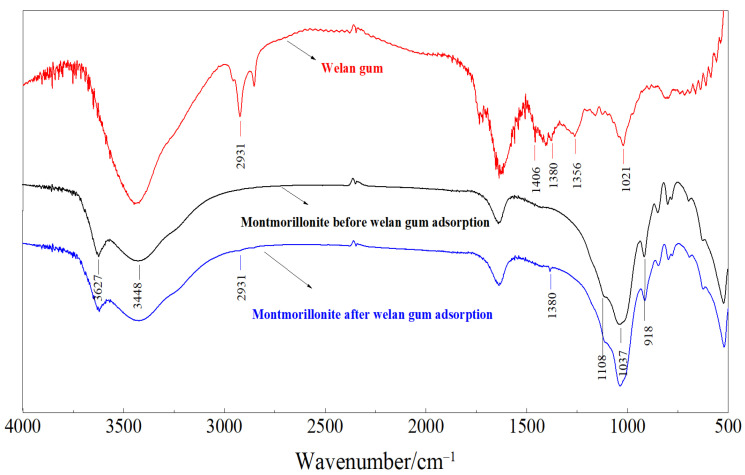
FT-IR spectra of welan gum and montmorillonite before and after welan gum adsorption.

**Figure 8:**
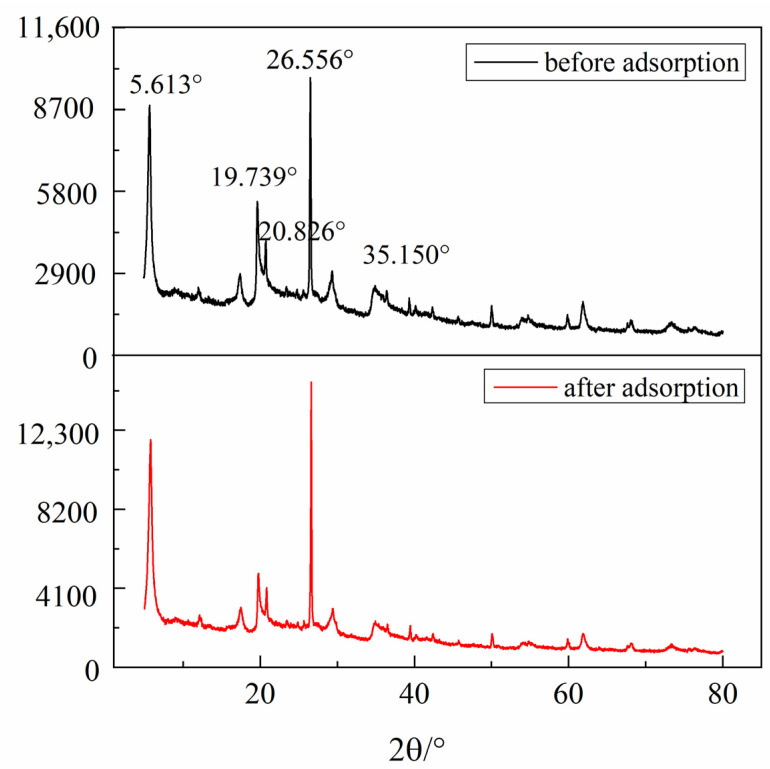
XRD spectra of montmorillonite before and after welan gum adsorption.

**Figure 9:**
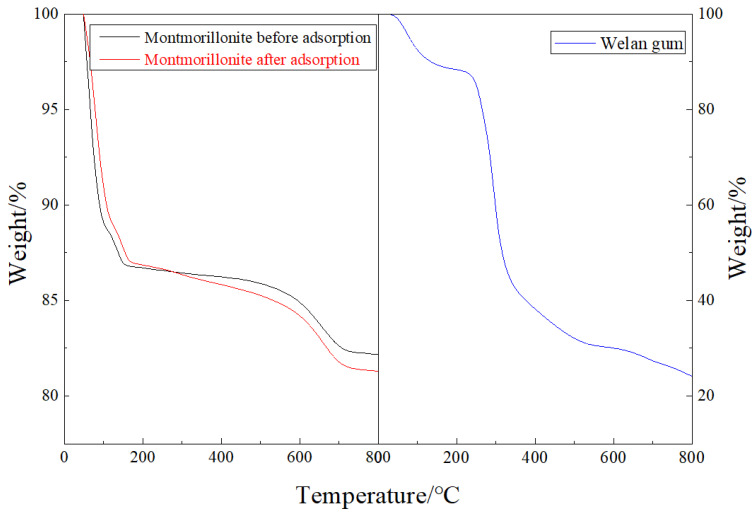
Thermalgravimetric curves of welan gum and montmorillonite before and after welan gum adsorption.

**Figure 10:**
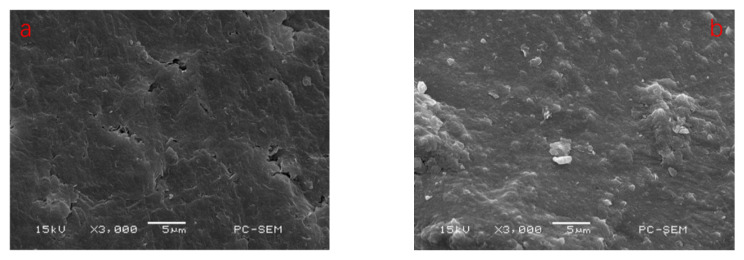
SEM images of montmorillonite before and after welan gum adsorption. (**a**) Before adsorption; (**b**) After adsorption.

In order to clarify the adsorption mechanism of welan gum on montmorillonite, it is necessary to study the physical and chemical changes of montmorillonite before and after adsorption of welan gum. Therefore, the functional groups, the weight loss rate, the changes of crystal spacing, and the micromorphology of the montmorillonite samples before and after welan gum adsorption were studied by using the Fourier transform infrared spectrometer (abbreviated as FT-IR, Nicolet is50, Thermo Fisher, Waltham, MA, USA), synchronous thermal analyzer (labsys Evo, Setaram, France), X-ray diffractometer (apex Ⅱ duo, Bruker, Bremen, Germany), and field emission scanning electron microscope (abbreviated as SEM, Supra 55, Zeiss, Berlin, Germany), respectively. The results are shown in Figure 7, Figure 8, Figure 9 and Figure 10.

#### 3.2.1. FT-IR

As can be seen from Figure 7, the red curve is the infrared spectrum curve of welan gum; the absorption peaks at 1406 cm^−1^, 1380 cm^−1^, 1356 cm^−1^, and 1201 cm^−1^ are the variable angle vibration of the C-H band. The absorption peaks at 2931 cm^−1^ belong to the adsorption peak of methylene -CH_2_-. The mentioned peaks together form the characteristic peaks of carbohydrate. 

The black curve is the infrared spectrum curve of montmorillonite that before welan gum adsorption. It has absorption peaks at 3627 cm^−1^, 3448 cm^−1^, 1108 cm^−1^, 1037 cm^−1^, and 918 cm^−1^. The peaks at wavenumber of 3627 cm^−1^ belong to the stretching vibration of Si-OH. The peaks at wavenumber of 3448 cm^−1^ belong to the stretching vibration of hydroxyl groups of water in montmorillonite. The peaks at wavenumbers of 1108 cm^−1^ belong to the stretching vibration of the Si-O bond. The absorption peaks at 1037 cm^−1^ and 918 cm^−1^ are the characteristic peaks of the asymmetric vibration of Si-O-Si and the stretching vibration peak of Al-O, which correspond to Si-O tetrahedron and Al-O octahedron in montmorillonite, respectively.

For the montmorillonite that after welan gum adsorption, new absorption peaks at 1380 cm^−1^ appeared, which belonged to the vibration of C-H band and were the characteristic peaks of carbohydrate. In addition, there were no new characteristic peaks generated, indicating that no new functional groups were generated during the adsorption process. 

#### 3.2.2. XRD

It can be seen from Figure 8 that for the montmorillonite sample before welan gum adsorption, 2θ = 6.06°, 19.92°, 22.01°, 35.78°, and 62.06° are the characteristic diffraction peaks of montmorillonite. The sharp and symmetrical diffraction peaks of the (001) plane indicate that the montmorillonite had orderly structure and good crystallinity. For the sample after welan gum adsorption, the value of 2θ did not change. This indicates that the crystal spacing of montmorillonite did not change. The results show that the adsorption of welan gum did not change the interlayer structure of montmorillonite, and welan gum is only adsorbed on the surface of montmorillonite. The reason is that welan gum is a macromolecule, and there is no ion exchange between montmorillonite and welan gum. Therefore, the adsorption only occurs on the surface of montmorillonite.

#### 3.2.3. TGA 

As can be seen from Figure 9, with the increase in temperature, there are two obvious weight loss stages for both the montmorillonite samples before and after welan gum adsorption. The first stage is the temperature range of 20 °C–300 °C, and the other stage is the temperature range of 300 °C–800 °C. When the temperature is lower than 300 °C, the weight loss rates of the two kinds of montmorillonite are basically the same. When the temperature is higher than 300 °C, the weight loss rate of the montmorillonite sample without welan gum adsorption (17.82%) is less than that of the montmorillonite sample with welan gum adsorption (18.77%). For welan gum, as the temperature increases, its weight loss mainly occurs within the temperature range of 20 °C–500 °C, as high as 70%. A coincidence is that the temperature range characterized by the obvious weight loss rate of welan gum was almost the same as the temperature range of the montmorillonite with adsorbed welan gum: exhibiting an obvious weight loss rate. This shows that the welan gum was adsorbed on the montmorillonite.

#### 3.2.4. SEM

As can be seen from Figure 10, the left image shows the surface of montmorillonite before welan gum adsorption, and the right image shows the surface of montmorillonite after welan gum adsorption. The surface of montmorillonite before adsorption is relatively smooth and there were small crackles, while the surfaces of the samples with the adsorbed welan gum were covered by a film, and the cracks were filled by the film material. By comparison, it can be concluded that the welan gum was adsorbed on the surface of montmorillonite.

### 3.3. Influencing Factors for Adsorption Behavior

#### 3.3.1. Effect of Temperature

**Figure 11:**
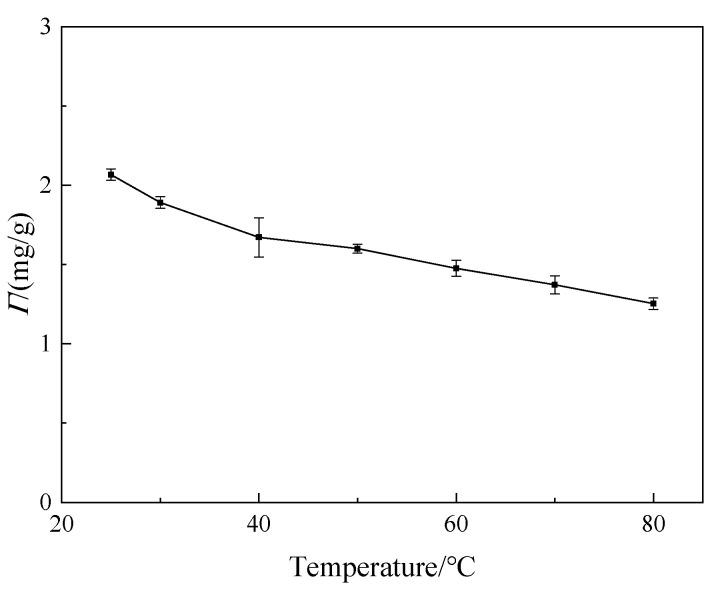
Effect of temperature on the adsorption of welan gum on montmorillonite.

The effect of temperature on the adsorption of welan gum on montmorillonite is shown in Figure 11.

As can be seen from Figure 11, with the increase in temperature, the adsorption capacity of welan gum on montmorillonite decreased gradually. It can be explained as follows: the adsorption of welan gum on montmorillonite is an exothermic reaction. It is not conducive to the transfer of the adsorption heat when the temperature increases. Therefore, the adsorption capacity decreases with the increase in temperature 33. In addition, when the temperature increases, the kinetic energy of the adsorbed welan gum molecules increases, meaning that the adsorbed welan gum may be desorbed. At the same time, the remanent molecules become difficult to adsorb onto the montmorillonite due to the increase in the kinetic energy. Therefore, the amount of adsorbed welan gum becomes less than the amount of desorbed welan gum, showing a decrease in the adsorption amount. Furthermore, the adsorption of welan gum on montmorillonite is a kind of weak physical adsorption. With the increase in temperature, the van der Waals force between montmorillonite and welan gum decreases, so the interaction between them weakens, resulting in the decrease in adsorption capacity [34].

#### 3.3.2. Effect of pH Value

**Figure 12:**
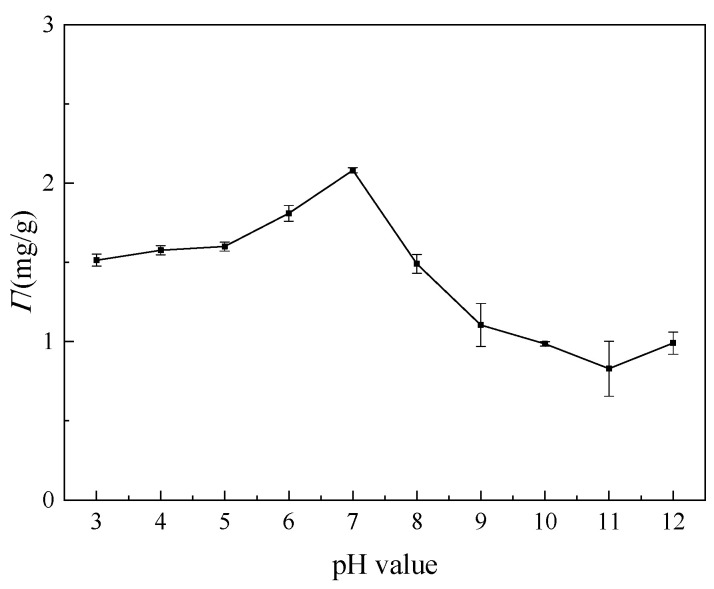
Effect of pH value on the adsorption of welan gum on montmorillonite.

The effect of pH value of the solution on the adsorption of welan gum on montmorillonite is shown in Figure 12.

As can be seen from Figure 12, the adsorption capacity in the neutral environment (pH = 7) is the largest. In both acidic and alkaline environments, the adsorption capacity is decreased, and the stronger the acidity or alkalinity is, the smaller the adsorption capacity is. When the pH value is in the range of 3–7, the adsorption capacity increases gradually. This is because there is a large amount of H^+^ in the acidic solution, which will affect the adsorption of welan gum on montmorillonite: the existence of H^+^ has a competitive relationship with the welan gum, and the adsorption capacity of welan gum is reduced because of the preference of montmorillonite to H^+^; with the increase in pH value, the content of H^+^ gradually decreases, resulting in an increase in the adsorption capacity of welan gum on montmorillonite [32]. 

In addition, when the pH value is in the range of 7–12, the adsorption capacity decreases gradually, and the decrease rate is larger than that in the acidic environment [33]. The reason is that both the montmorillonite and the welan gum are negatively charged, so the adsorption capacity is reduced due to electrostatic repulsion [35]. In addition, the stronger the alkalinity of the solution (that is, the higher of the pH value) is, the more negative charge on the montmorillonite is, and the greater the repulsion between the montmorillonite and the welan gum is, which leads to the lower adsorption capacity.

#### 3.3.3. Effect of Inorganic Salt

**Figure 13:**
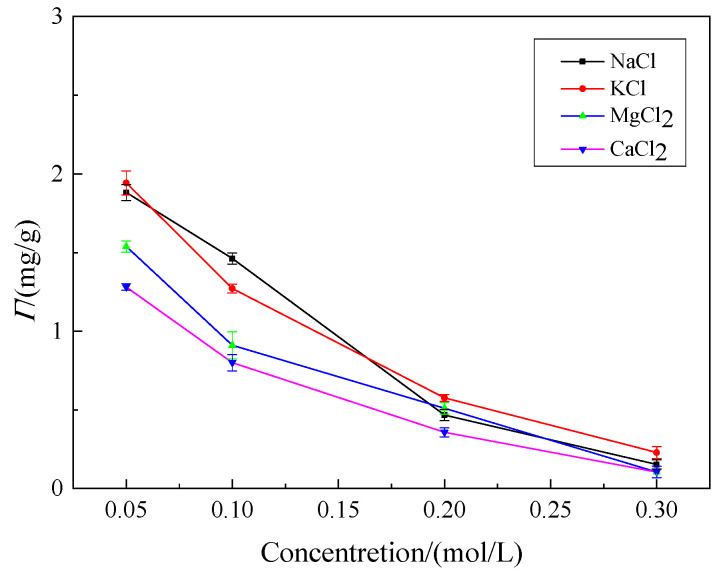
Effect of inorganic salt on welan gum adsorption on montmorillonite.

The effect of kind of inorganic salt, as well as its concentration, on the adsorption of welan gum on montmorillonite is shown in Figure 13.

As can be seen from Figure 13, the addition of inorganic salt has a great influence on the adsorption. With the increase in inorganic salt content, the adsorption capacity of welan gum on montmorillonite decreases sharply. This phenomenon can be explained as follows. Montmorillonite is negatively charged, when there are cations (Na^+^, K^+^, Mg^2+^, Ca^2+^) in welan gum solution, these cations will be preferentially adsorbed by montmorillonite, resulting in a reduction in the adsorption capacity of welan gum. 

In addition, these cations can compress the diffused double-layer structure of the colloidal particles formed by the hydration and expansion of montmorillonite, resulting in the reduction in the volume of montmorillonite. This change reduces the area used for welan gum adsorption, which ultimately affects the adsorption capacity [36].

Furthermore, when there are a large number of inorganic cations in the solution, it will promote the curling of the macromolecular chain of the welan gum, and reduce the contact points and forces between the welan gum molecules and the montmorillonite. Therefore, the number of the desorbed welan gum molecules is larger than that of the adsorbed molecules within the same time, resulting in a reduction in the adsorption capacity of welan gum on montmorillonite at the macro level [37].

It also can be seen from Figure 11 that the reduction rate of the adsorption capacity in the solution containing Mg^2+^ and Ca^2+^ was larger than that in the solution containing Na^+^ and K^+^. This is because Mg^2+^ and Ca^2+^ are divalent cations, and they have stronger effects mentioned above than Na^+^ and K^+^. Therefore, the adsorption capacity of welan gum on montmorillonite in the solution containing Mg^2+^ and Ca^2+^ was lower than Na^+^ and K^+^. 

In conclusion, the external environment has a great impact on the adsorption performance of montmorillonite. The adsorption capacity of welan gum on montmorillonite decreases with the increase in temperature, acidity, or alkalinity of the welan gum solution, and inorganic salt content in the solution. Borrowing these rules can help reduce reservoir damage caused by the adsorption of welan gum on montmorillonite when welan gum is used in polymer flooding.

## 4. Conclusions

In polymer-flooding-enhanced oil recovery technology, welan gum can be a viscosity promoter for the oil displacement in the fluid, and can be adsorbed on the surface of clay minerals (montmorillonite). The maximum adsorption capacity was 2.07 mg/g, and the adsorption equilibrium time was 10 min. In the adsorption process, no chemical reaction occurred, and no new functional groups were generated. The adsorption mainly occurred on the surface of montmorillonite, and its crystal spacing was not changed after welan gum adsorption. 

The temperature, pH value, and the inorganic salt content of the flooding fluid affected the adsorption capacity of welan gum on montmorillonite. The adsorption capacity reduced by 39.6% when the temperature increased from 25 °C to 80 °C. When the pH value was 3 or 12, the adsorption capacity decreased by 27.5% or 52.4%, respectively, compared with that when the pH was 7. The higher the inorganic salt content, the greater the reduction rate of the adsorption capacity. Taking CaCl_2_ as an example, when the concentration increased from 0 to 0.3 mol/L, the adsorption capacity reduced by 95%.

From the above research results of the influence of temperature, pH value, and inorganic salts on the adsorption capacity, it can be seen that, in the reservoir conditions—which are usually high-temperature, high-salt environments—the adsorption capacity of welan gum on the surface of montmorillonite will be greatly reduced. That is, montmorillonite is not the main factor causing the adsorption and retention of welan gum in the reservoir.

## Figures and Tables

**Table 1:** Typical studies of microbial polysaccharide used in polymer flooding.

No.	Polymer Types	Key Findings	Ref.
1	Xanthan gum, HPAM	Xanthan has good performance at 80 °C, but weakens when the temperature exceeds 100 °C.	Li et al. [13]
2	Xanthan gum, HPAM	Xanthan gum and HPAM have synergistic effects.	Cai [14]
3	Xanthan gum, Guanidine gum	XG is affected little by acid, alkali, and bacterial environments.	Ming et al. [15]
4	Xanthan gum	It had good salt tolerance, and could be used for enhancing heavy oil recovery.	Xu et al. [16]
5	Xanthan gum, welan gum	Welan gum had better viscosity-retention rate (72%) than xanthan gum (50%) at 75 °C.	Xu et al. [17]
6	Welan gum, dieter gum, xanthan gum, and HPAM	Welan gum has better temperature resistance than xanthan gum, resulting better ability in improving oil recovery.	Zhou et al. [18]
7	Welan gum, HPAM	Welan gum solution is pseudoplastic fluid with good viscoelasticity, and is affected little by sodium ion and potassium ion.	Ji et al. [20]
8	Welan gum	It is a good candidate for polymer flooding in reservoirs with high temperatures and high salt content.	Li et al. [21], Wang et al. [22]

## Data Availability

The data presented in this study are available on request from the corresponding author.
